# Self-reported learning-oriented GenAI use under perceived teacher-referenced normative tension in Chinese higher education

**DOI:** 10.3389/fpsyg.2026.1969743

**Published:** 2026-10-02

**Authors:** Zhenzhen Yang, Yong Yang, Wei Liu

**Affiliations:** 1 Automotive Engineering College, Weifang Vocational College, Weifang, Shandong, China; 2 General Education Center, Weifang Vocational College, Weifang, Shandong, China; 3 Graduate School, José Rizal University, Mandaluyong, Philippines

**Keywords:** generative artificial intelligence, higher education, learning-oriented GenAI use, normative uncertainty, perceived teacher-referenced normative tension, self-regulated learning

## Abstract

**Objective:**

The educational implications of generative artificial intelligence (GenAI) depend not only on whether students use these tools, but also on how they orient their use when acceptable-use expectations remain unsettled. This study examined whether perceived teacher-referenced GenAI normative tension (PNT) was associated with self-reported learning-oriented GenAI use (LOU) and whether LOU remained distinguishable from related academic experiences and learning tendencies in Chinese higher education.

**Methods:**

Cross-sectional survey data were collected from 1,004 students at three public universities. Covariance-based structural equation modelling was used to evaluate the proposed five-factor measurement representation and a theory-constrained six-path associational model. Internal split-sample measurement analyses, alternative measurement models, covariate-adjusted models, an ordinal-estimator sensitivity analysis, institution sensitivity analyses, a reverse focal specification, and exploratory structural extensions were also conducted.

**Results:**

The proposed five-factor measurement representation showed close fit and was reproduced in a held-out subsample. The focal PNT–LOU association was positive and statistically significant, while the remaining structural paths positioned LOU within a broader network of related academic experiences and learning tendencies. PNT was positively associated with self-reported LOU (β = 0.568, *p* < 0.001), and the model explained 32.2% of the variance in LOU. The focal association remained positive after adjustment for GenAI guidance clarity (β = 0.478, *p* < 0.001), demographic and institutional covariates (β = 0.570, *p* < 0.001), and use of an ordinal estimator. An observationally equivalent reverse specification produced the same global fit, indicating that temporal direction could not be identified. Exploratory extensions further indicated that the six-path specification did not exhaust the observed covariance structure, particularly for ACS.

**Conclusion:**

Perceived difficulty locating one’s GenAI practices relative to teacher-referenced expectations and self-reported learning-oriented GenAI use can coexist rather than functioning as opposite ends of a single continuum. However, the cross-sectional, same-source design does not establish that normative tension improves learning-oriented behaviour. Reverse direction, shared unmeasured characteristics, common-method inflation, social desirability, and legitimacy-oriented self-presentation remain plausible explanations.

## Introduction

The rapid expansion of generative artificial intelligence (GenAI) has created a governance challenge within higher education: students increasingly use GenAI for academic writing, information retrieval, brainstorming, and learning support, while institutions and educators continue to negotiate its educational opportunities, risks, and acceptable forms of use (Dwivedi et al., 2023). Systematic reviews have identified both growing educational use and recurring concerns about academic integrity, over-reliance, and inconsistent governance (Batista et al., 2024; Hon, 2026; Zhai et al., 2024). Student-centred research has similarly documented perceived educational benefits of GenAI alongside concerns about accuracy, academic integrity, and responsible use (Chan and Hu, 2023). Empirical studies in Chinese higher education likewise indicate that undergraduate students are incorporating GenAI into academic and writing-related practices while negotiating its educational value and appropriate use (Li et al., 2024; Kim et al., 2024). These developments make the central educational question not simply whether students use GenAI, but how they orient their use when acceptable-use expectations remain uncertain, inconsistent, or teacher-dependent.

Much of the existing GenAI literature has examined technology acceptance, behavioural intention, and adoption through frameworks such as the Technology Acceptance Model and the Unified Theory of Acceptance and Use of Technology (Venkatesh et al., 2003; Bali et al., 2024; Moradi, 2025). Recent reviews likewise identify adoption, use, academic integrity, and learning outcomes as prominent themes in higher-education research (Batista et al., 2024; Hon, 2026). These perspectives are informative for understanding whether and why students use GenAI, but they provide less direct evidence about how students position their own practices when teacher-referenced expectations remain uncertain or inconsistent. Related studies have considered social influence, trust, and cultural values in AI-related decision-making (Graf-Vlachy et al., 2018; Huang et al., 2019; Barnes et al., 2024). This literature explains whether and why students adopt GenAI, but leaves a different problem unresolved: what students report doing when they continue using GenAI without a stable teacher-referenced position from which to judge whether their own practices are acceptable. The present study provisionally conceptualises this experience as perceived teacher-referenced GenAI normative tension (PNT), defined as students’ reported difficulty locating their GenAI practices within a stable teacher-referenced standard of acceptable use. Its indicators address perceived disagreement, alignment uncertainty, unclear expectations, and inconsistency across teachers. PNT is treated as an initial, entirely student-reported operational representation rather than as an established construct or an objectively verified teacher–student discrepancy. Unlike general role ambiguity or institutional guidance uncertainty, PNT specifically concerns students’ perceived difficulty locating their own GenAI practices relative to teacher-referenced standards of acceptable use.

Ambiguous or inconsistently applied expectations may correspond with withdrawal, concealment, minimal compliance, or continued use described in academically defensible terms. Research on demanding and uncertain conditions provides a limited basis for questioning whether normative difficulty must necessarily correspond with disengagement (Cavanaugh et al., 2000; Crawford et al., 2010). The present study therefore examines the narrower possibility that perceived normative-positioning difficulty can coexist with stronger self-reported learning-oriented GenAI use. Because challenge and hindrance appraisals were not measured, the study does not classify PNT as a challenge stressor or propose a challenge-based psychological mechanism.

Self-Determination Theory suggests that students’ academic engagement is associated with autonomy- and competence-related experiences (Ryan and Deci, 2020). Reflective engagement and self-regulated learning have similarly been linked to motivational regulation, metacognitive monitoring, and independent learning strategies (Panadero, 2017; Schunk and Zimmerman, 2011). In GenAI-supported contexts, students who describe their use in more learning-oriented terms may also report stronger autonomy- and competence-related academic experience, self-regulated learning, and critical-evaluation tendencies because these self-reports concern related aspects of academically engaged learning (Dahri et al., 2024; Xia et al., 2023). These relationships are particularly relevant in Chinese higher education, where recent studies document growing student use of GenAI alongside continuing uncertainty about appropriate academic use (Li et al., 2024; Kim et al., 2024). In this setting, teacher-referenced expectations may constitute a particularly proximal source of normative information for students, although the present study does not directly compare the influence of teachers with that of institutions or peers. The Chinese public-university context therefore provides a relevant setting for examining perceived normative positioning while also limiting generalisation beyond the sampled institutions.

Recent reviews of GenAI in higher education indicate that a substantial part of the literature has focused on technology acceptance and use, academic integrity, learning outcomes, and concerns about over-reliance (Batista et al., 2024; Hon, 2026). These literatures establish the educational significance of GenAI use but leave less clearly resolved how students position their own practices when teacher-referenced expectations are uncertain or inconsistent. Emerging empirical evidence further suggests that students’ AI-related academic conduct may be shaped by perceived ethical and instructor-referenced norms rather than by institutional policy awareness alone (Lund et al., 2025; Huang et al., 2025). However, research on normative uncertainty and research on cognitively engaged or learning-oriented GenAI use have largely developed as separate strands. It therefore remains unclear whether students who perceive greater teacher-referenced normative difficulty necessarily report less learning-oriented use, or whether these experiences can coexist. A further unresolved issue is whether GenAI-specific learning-oriented use remains empirically distinguishable from broader self-regulated learning and related academic tendencies. The present study addresses these linked questions by distinguishing the normative conditions students perceive from the orientation they report adopting toward GenAI-supported learning. Against this background, the focal research question is whether perceived teacher-referenced normative tension is positively associated with self-reported learning-oriented GenAI use. The study tests whether normative-positioning difficulty and learning-oriented use can coexist rather than functioning as opposite ends of a single continuum. The remaining constructs serve a secondary positioning function: ACS, SRL, and CT locate LOU within a broader network of related academic experiences and learning tendencies, while GGC is used to examine whether PNT remains distinguishable from perceived guidance clarity. The study therefore contributes primarily to construct differentiation and to identifying a contestable cross-sectional association, without claiming temporal ordering, adaptive response, or observed GenAI behaviour.

### Theoretical background and hypothesis development

#### Perceived teacher-referenced GenAI normative tension and learning-oriented GenAI use

The rapid adoption of GenAI has created educational environments in which acceptable-use expectations may differ across teachers, courses, and institutions. Debate over appropriate governance has ranged from conditional permission and responsible-use guidance to arguments that restrictive or prohibitive approaches may be ethically justified in particular educational circumstances (de Fine Licht, 2024). Although prior studies have largely examined GenAI through usefulness and technology-acceptance perspectives (Bali et al., 2024; Moradi, 2025), less attention has been given to how students respond when they cannot determine whether their own practices align with teacher-referenced expectations. Building on the definition introduced above, PNT is treated here as a provisional student-reported normative-positioning construct rather than an objectively verified teacher–student discrepancy or a general measure of institutional guidance clarity.

Ambiguous or inconsistently applied expectations may correspond with several student responses, including reduced use, concealment, minimal compliance, or continued GenAI use organised around practices that students regard as academically defensible. The challenge-hindrance perspective provides a limited basis for questioning whether demanding or uncertain conditions necessarily correspond with withdrawal (Cavanaugh et al., 2000; Crawford et al., 2010). However, because the present study did not measure challenge or hindrance appraisal, this perspective is not used to classify PNT as a challenge stressor or to claim that normative tension is inherently beneficial.

The directional expectation proposed here is narrower. Students who continue using GenAI while experiencing difficulty locating their practices within teacher-referenced expectations may place greater emphasis on forms of use that preserve visible cognitive involvement, such as concept clarification, evaluation of generated content, integration with independent reasoning, and identification of gaps in understanding. These practices are more compatible with conventional academic expectations concerning student agency and intellectual responsibility than direct answer substitution or uncritical task completion. Accordingly, students reporting greater teacher-referenced normative-positioning difficulty may also report stronger learning-oriented GenAI use.

This contestable prediction concerns a directional between-student association rather than a universal response or causal compensatory mechanism. Several alternative explanations remain possible. Students who already use GenAI in more reflective ways may be more attentive to disagreement or inconsistency in teacher expectations. Both reports may also reflect academic self-efficacy, normative attentiveness, a general tendency to describe one’s engagement positively, or legitimacy-oriented self-presentation. The hypothesis therefore tests whether the proposed positive association is present, not why it occurs or which temporal direction generated it.

*H1:* Perceived teacher-referenced GenAI normative tension (PNT) is positively associated with self-reported learning-oriented GenAI use (LOU).

#### Self-reported learning-oriented GenAI use as a GenAI-specific correlate

Self-reported learning-oriented GenAI use differs from convenience-oriented or task-substitutive use because its items emphasise continued cognitive involvement in interpreting, evaluating, and integrating AI-generated information. This distinction is educationally important because evidence on GenAI-supported learning is mixed. Productive use may support access to explanations, feedback, and task performance, whereas over-reliance can reduce independent cognitive or metacognitive processing (Zhai et al., 2024; Bauer et al., 2025). LOU therefore captures a self-reported orientation toward maintaining cognitive involvement during GenAI use rather than assuming that GenAI use itself is inherently educationally beneficial. Although LOU is related to self-regulated learning, the constructs differ in scope: LOU captures students’ reported GenAI-specific practices such as concept clarification, feedback interpretation, and output evaluation, whereas SRL captures domain-general planning, monitoring, and adjustment across academic tasks. The distinction is conceptual and empirical, but LOU remains a self-report measure rather than direct evidence of actual use behaviour.

LOU may also be positively associated with combined autonomy- and competence-related academic experience (ACS). Self-Determination Theory provides a limited conceptual rationale for considering autonomy- and competence-related academic experience as a correlate of engaged learning (Ryan and Deci, 2020). Students who report more active and self-directed GenAI engagement may also report more positive autonomy- and competence-related experiences in their broader academic learning. However, because the present ACS measure did not empirically distinguish autonomy from competence and assessed general academic experience rather than a GenAI-specific state, the proposed LOU–ACS association is not treated as a formal test of Self-Determination Theory or as evidence of a psychological-need mechanism.

LOU is also relevant to self-reported critical-evaluation tendencies (CT). Because GenAI systems may generate biased or inaccurate outputs, students who describe their use as learning-oriented may also report questioning assumptions, comparing explanations, assessing evidence, and refining arguments during academic tasks (Urban et al., 2024; Daniel et al., 2025). This association concerns aligned self-reported tendencies and does not establish improvement in general critical-thinking ability. Accordingly, the following hypotheses are proposed.

*H2:* Self-reported learning-oriented GenAI use (LOU) is positively associated with combined autonomy- and competence-related academic experience (ACS).

*H3:* Self-reported learning-oriented GenAI use (LOU) is positively associated with self-regulated learning (SRL).

*H4:* Self-reported learning-oriented GenAI use (LOU) is positively associated with self-reported critical-evaluation tendencies (CT).

#### Combined autonomy- and competence-related academic experience

Autonomy- and competence-related academic experiences provide a limited motivational lens for examining how self-reported learning-oriented engagement is associated with broader learning tendencies. Self-Determination Theory treats autonomy and competence as distinct psychological needs associated with academic motivation and engagement (Ryan, 2017; Ryan and Deci, 2020). The present five-item measure did not empirically distinguish these dimensions, so ACS is used only as a combined academic correlate. It should not be interpreted as evidence that autonomy and competence are theoretically identical or as a formal test of the multidimensional need structure proposed by Self-Determination Theory.

Within GenAI-supported learning contexts, students reporting more positive autonomy- and competence-related academic experience may also report stronger self-regulation and critical-evaluation tendencies. Such covariation may reflect connected aspects of academic engagement, general positive self-presentation, or other unmeasured student characteristics. The proposed relationships are therefore associational and do not imply that ACS temporally precedes or causes SRL or CT.

*H5:* Combined autonomy- and competence-related academic experience (ACS) is positively associated with self-regulated learning (SRL).

*H6:* Combined autonomy- and competence-related academic experience (ACS) is positively associated with self-reported critical-evaluation tendencies (CT).

### Research model

Figure 1 presents the theoretically specified associational model. H1 constitutes the focal theoretical test and concerns the relationship between PNT and self-reported LOU. H2–H6 position LOU within a nomological network of related academic experiences and learning tendencies. Specifically, LOU is expected to be associated with ACS, SRL, and CT, while ACS is further expected to be associated with SRL and CT. These paths are not proposed as a fully identified psychological process or an exhaustive representation of the covariance structure. Direct associations from PNT to ACS, SRL, and CT were therefore examined separately as exploratory structural extensions. All directional arrows represent theoretical specification rather than temporal or causal precedence.

**Figure 1:**
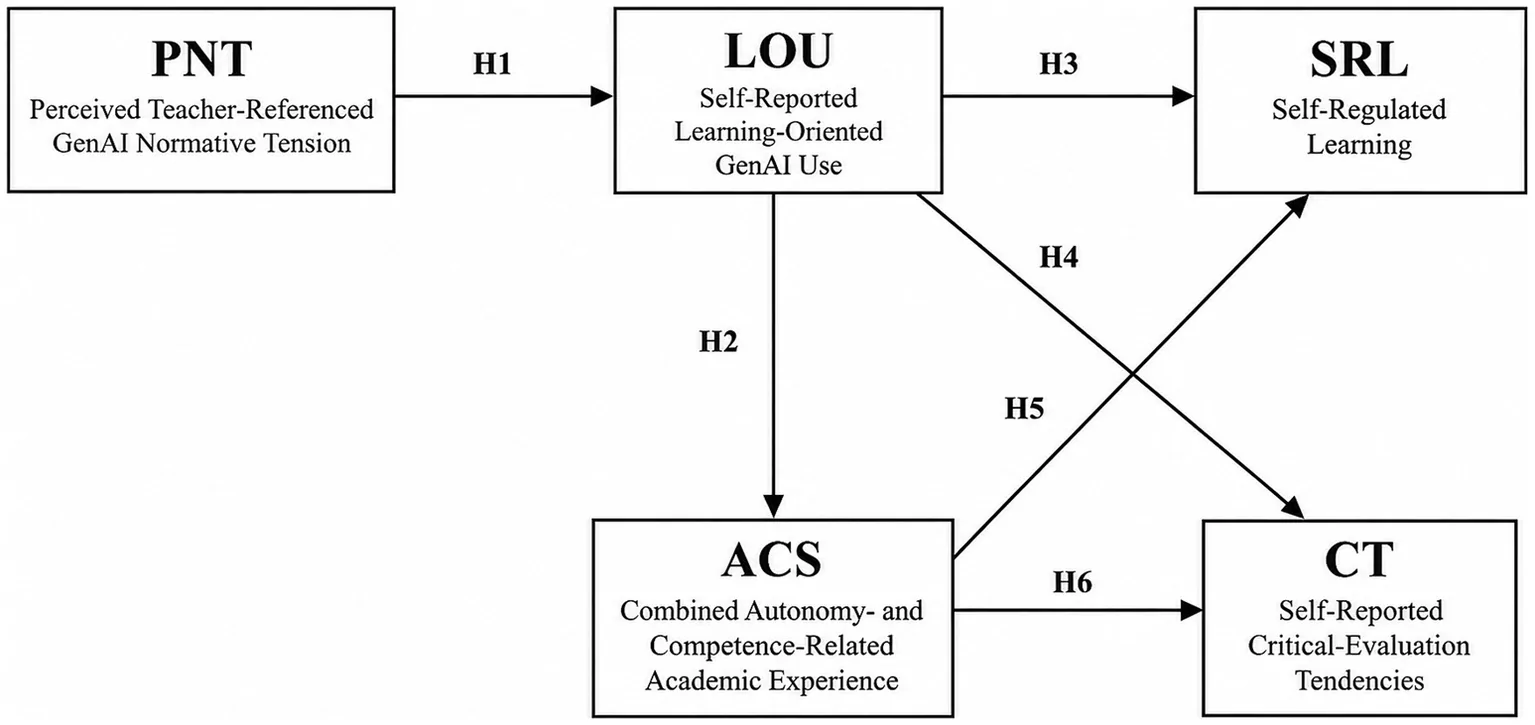
Hypothesised associational model. H1 denotes the focal association between PNT and LOU. H2–H6 position LOU within a broader nomological network of related academic experiences and learning tendencies. LOU represents self-reported learning-oriented GenAI use, ACS represents combined autonomy- and competence-related academic experience, and CT represents self-reported critical-evaluation tendencies. Directional arrows represent theory-constrained model specification and do not establish temporal or causal effects. The model is not presented as an exhaustive covariance structure; exploratory direct paths from PNT are omitted for clarity.

## Methods

### Research design

This study employed a quantitative cross-sectional design to examine associations among perceived teacher-referenced GenAI normative tension (PNT), self-reported learning-oriented GenAI use (LOU), combined autonomy- and competence-related academic experience (ACS), self-regulated learning (SRL), and self-reported critical-evaluation tendencies (CT) among Chinese university students. PNT was operationalised as a student-reported teacher-referenced normative-positioning difficulty, consistent with the provisional conceptual definition developed above. Because the data were collected at a single time point, the directional model represented a theoretically specified ordering of associations and did not establish temporal precedence or causal effects. Participation was anonymous and voluntary, respondents were informed that there were no right or wrong answers, and responses were collected solely for research purposes. These procedures may reduce evaluation apprehension but cannot eliminate common-method covariance.

### Participants and procedure

Participants were recruited from three public universities in mainland China during the 2025–2026 academic year using purposive institutional selection and convenience-based student recruitment. During scheduled classes, instructors shared the survey link and QR code with students, and the questionnaire was administered online through Wenjuanxing. Participation was voluntary and was not linked to academic grades or course assessment. Because the total number of students who received or viewed the survey invitation was not systematically recorded, a conventional response rate could not be calculated. A total of 1,020 student questionnaires were initially collected. Sixteen responses were removed because they failed an embedded attention-check item, resulting in a final sample of 1,004. All participants in the final sample reported prior academic or learning-related GenAI use: three reported using GenAI rarely, 621 sometimes, and 380 often, while no valid respondent selected “never.” GenAI-use frequency was not a formal eligibility criterion; thus, the absence of non-users reflects the composition of the convenience sample rather than deliberate exclusion.

The final sample consisted of 491 male students (48.9%) and 513 female students (51.1%). Of these, 361 (36.0%) were enrolled in STEM-related programmes and 643 (64.0%) in humanities and social-science programmes. The sample included 490 sophomores (48.8%) and 514 juniors (51.2%) and was distributed across Institution A (*n* = 331), Institution B (*n* = 306), and Institution C (*n* = 367). Table 1 summarises the demographic characteristics of the final student sample. The restriction to sophomore and junior students reflected the classes available to the research team during the data-collection period rather than an *a priori* exclusion of first- or fourth-year students. The disciplinary imbalance and restriction to these two year levels should therefore be considered when interpreting sample representativeness and generalisability.

**Table 1:** Demographic characteristics of student participants (*N* = 1,004).

Variable	Category	Frequency	Percentage
Gender	Male	491	48.9
	Female	513	51.1
Academic discipline	STEM	361	36.0
	Humanities & social sciences	643	64.0
Year level	Sophomore	490	48.8
	Junior	514	51.2
Institution	Institution A	331	33.0
	Institution B	306	30.5
	Institution C	367	36.5

A separate unmatched survey was completed by 148 faculty members from the same three universities. These data were not linked to individual students, courses, classrooms, or instructors and did not assess teacher-side equivalents of the student PNT items. They were therefore used only to provide group-level descriptive context and were not included in the focal measurement or structural models. The five-factor measurement model contained 60 free parameters, corresponding to approximately 16.7 observations per free parameter in the student sample (*N* = 1,004). This exceeds commonly cited rule-of-thumb ratios used as preliminary indicators of sample adequacy in structural equation modelling, although such ratios should not be treated as sufficient evidence of adequate statistical power because required sample size depends on model complexity, factor loadings, estimator, and other design characteristics (Kline, 2016; Wolf et al., 2013). Sample adequacy was therefore considered together with parameter precision and the stability of the measurement structure in the held-out validation subsample. Data were collected between December 2025 and March 2026. Before completing the questionnaire, all respondents were informed about the purpose of the study and provided electronic informed consent. Respondents could discontinue participation at any stage without penalty.

### Measures

All student questionnaire items employed a five-point Likert scale ranging from 1 (“strongly disagree”) to 5 (“strongly agree”). The instrument was initially drafted in English and subsequently translated into simplified Chinese using the forward-backward translation procedure proposed by Brislin (1970). The translated questionnaire was reviewed by six experts specialising in educational technology, higher education pedagogy, and GenAI-related educational policy to ensure linguistic clarity and contextual appropriateness. Full item wording and original questionnaire codes for the student measures included in the focal measurement model and the GGC robustness extension are reported in Supplementary Table S1.

A pilot test involving 42 university students was conducted before the main survey. Participants commented on item clarity, comprehension, and whether the PNT items conveyed teacher-referenced discrepancies, unclear expectations, or inconsistently applied standards concerning acceptable GenAI use. No PNT items were removed during the pilot, and all five indicators were retained. Minor wording revisions were made before the main survey to make references to teachers and teacher expectations more explicit, to clarify that the items concerned acceptable GenAI use in academic work, and to reduce ambiguity with general AI anxiety or institution-level guidance. The factorial structure, reliability, convergent validity, and discriminant validity of the measures were subsequently evaluated using latent-variable measurement-model analyses.

Perceived teacher-referenced GenAI normative tension (PNT). PNT was assessed using five contextually adapted items informed by established measures of role ambiguity, role conflict, and goal/process clarity (Rizzo et al., 1970; Sawyer, 1992). The adaptation focused on students’ perceived difficulty locating their own GenAI practices within a stable teacher-referenced standard of acceptable use. The items addressed perceived disagreement with teacher-attributed expectations, uncertainty about alignment, unclear expectations, and inconsistency across teachers. Example items included “My teachers and I seem to hold different views about appropriate GenAI use in academic work” and “I am often unsure whether my use of GenAI aligns with my teachers’ expectations.” The measure was entirely student-reported and did not incorporate matched teacher responses. It therefore represents perceived teacher-referenced normative tension rather than an objectively verified teacher-student discrepancy.

The items were administered under the working label “Perceived Teacher–Student Misalignment” and retained their original questionnaire codes (PTM1–PTM5). The reporting label “perceived teacher-referenced GenAI normative tension” was finalised after data collection to reflect more accurately that the items assessed students’ perceptions of teacher-referenced expectations rather than matched teacher–student discrepancies; neither item wording nor the analytical specification was altered. The five indicators were modelled as an initial context-specific representation of related normative-positioning experiences. Two indicators concerning unclear or inconsistent teacher expectations are conceptually adjacent to guidance clarity. Accordingly, PNT was distinguished from GGC by its focus on students’ difficulty positioning their own practices relative to teacher-referenced expectations, whereas GGC assessed the perceived clarity and consistency of formal or course-level guidance. The constructs were therefore expected to be related rather than completely independent. Full item wording and adaptation information are provided in Supplementary Table S1.

Learning-oriented GenAI use (LOU). LOU was assessed using five context-specific self-report items developed for the present study as an initial operationalisation of students’ reported emphasis on cognitively active and learning-focused forms of GenAI use. The items emphasised conceptual understanding, feedback interpretation, output evaluation, and integration with independent reasoning rather than answer substitution or task-completion efficiency. Example items included “I use GenAI to deepen my understanding of complex concepts rather than simply obtain answers” and “I integrate GenAI feedback with my own reasoning.” All five items referred specifically to GenAI-supported engagement and did not assess general planning, goal-setting, or task-management processes. Because the items describe academically desirable practices, responses may partly reflect preferred or self-legitimating descriptions of GenAI use. The measure captures students’ reported learning-oriented use orientation rather than directly observed GenAI behaviour and may be influenced by recall, social desirability, or legitimacy-oriented self-presentation.

Combined autonomy- and competence-related academic experience (ACS). ACS was assessed using five items adapted from autonomy- and competence-related content within the Basic Psychological Need Satisfaction framework (Chen et al., 2015). The items assessed general academic experiences and did not assess relatedness. Because autonomy and competence are theoretically distinct, a constrained two-factor specification separating the three autonomy items from the two competence items was compared with the combined one-factor specification. Although the two-factor model showed superficially close global fit, the estimated latent correlation exceeded unity (r = 1.029), producing an inadmissible solution. The two dimensions could not be empirically distinguished with the present five-item measure, so the one-factor representation was retained as a combined academic correlate. This pragmatic decision does not imply that autonomy and competence are theoretically identical or constitute a formal test of the multidimensional need structure proposed by Self-Determination Theory. Example items included “In my learning, I feel I can make my own choices” and “I feel confident in solving academic problems.” The five items retained their original NS1–NS5 questionnaire codes. Item wording and analytical specification were unchanged after data collection, while the reporting label “combined autonomy- and competence-related academic experience” (ACS) was finalised *post hoc* to reflect the fact that the abbreviated measure did not empirically distinguish autonomy from competence.

Self-regulated learning (SRL). SRL was assessed using five items developed for the present study with reference to the goal-setting, planning, monitoring, strategy-adjustment, and reflection processes described by Schunk and Zimmerman (2011) and Panadero (2017). Example items included “I set clear learning goals before starting academic tasks” and “After completing a task, I reflect on how I could improve my learning.” The items assessed domain-general learning-management processes and did not refer to GenAI or any other specific technology.

Critical thinking (CT). CT was assessed using five items developed for the present study to capture students’ reported tendencies to evaluate information reliability, consider alternative viewpoints, examine evidence, identify weaknesses in arguments, and reflect on their own assumptions. Example items included “I usually question whether information is reliable before accepting it” and “I reflect on my own assumptions when solving academic problems.” The measure captures self-reported critical-evaluation tendencies rather than demonstrated performance on a critical-thinking task.

GenAI guidance clarity (GGC). GGC was measured using five items assessing students’ perceptions of the clarity and consistency of institutional and instructional guidance regarding acceptable GenAI use. Example items included: “My institution provides clear guidance on acceptable GenAI use” and “My courses provide consistent rules about acceptable GenAI use.” GGC was included as an adjacent construct against which the empirical distinctiveness and incremental association of PNT could be assessed. GGC captures the perceived clarity and consistency of formal or course-level guidance, whereas PNT captures students’ difficulty positioning their own practices relative to teacher-referenced expectations. GGC was not part of the hypothesised six-path model but was added in a robustness model predicting LOU and ACS.

### Data analysis

Descriptive statistics, demographic summaries, Cronbach’s alpha, and supplementary teacher–student aggregate comparisons were conducted using SPSS 26. Covariance-based structural equation models and model-based measurement-quality analyses were conducted in R version 4.6.1 using the lavaan package version 0.6–21. Maximum-likelihood (ML) estimation was used for the primary measurement and structural models unless otherwise stated, while the ordinal-item sensitivity model was estimated using diagonally weighted least squares (DWLS). Before analysis, the student dataset was screened for missing values, out-of-range responses, duplicate identifiers, and attention-check failure. The study was not preregistered. The six structural paths constituted the *a priori* theoretical specification, whereas additional direct paths from PNT to ACS, SRL, and CT were examined *post hoc*. Alternative measurement specifications, GGC and covariate adjustments, DWLS estimation, reverse specification, institution-sensitivity analyses, and the split-sample analysis were treated as measurement, robustness, sensitivity, or internal-replication analyses rather than additional confirmatory hypothesis tests.

The analysis proceeded in four stages. First, the hypothesised five-factor measurement model comprising PNT, LOU, ACS, SRL, and CT was evaluated using χ^2^, CFI, TLI, RMSEA with its 90% confidence interval, and SRMR, together with reliability, convergent-validity, discriminant-validity, and residual diagnostics. Alternative specifications were examined for PNT and ACS, and the focal measurement model was compared with theoretically relevant collapsed-factor alternatives and a six-factor extension including GGC. Second, the theoretically specified six-path structural model was estimated. Third, post-hoc exploratory models added direct paths from PNT to ACS, SRL, and CT to assess whether the focal specification omitted meaningful residual associations. Fourth, robustness and sensitivity analyses included adjustment for GGC and demographic and institutional covariates, DWLS estimation, a reverse focal specification, and institution-sensitivity analyses. Formal measurement invariance across institutions was not tested; therefore, the institution-specific and leave-one-institution-out analyses were interpreted as sensitivity checks rather than evidence of cross-institution measurement equivalence. Standardised indirect products were estimated using 5,000 bootstrap resamples and treated as algebraic model implications rather than evidence of temporal mediation. A split-sample calibration and validation analysis provided an internal measurement-replication check. Detailed supplementary specifications are reported in the Supplementary Materials, and Table 2 summarises the analytical status of the primary and additional analyses.

**Table 2:** Analytical status of the primary and additional analyses.

Analysis	Specification	Analytical status
Primary structural model	Six hypothesised paths, H1–H6	*A priori* theoretical specification
Exploratory extension 1	Additional PNT → ACS path	*Post hoc* exploratory
Exploratory extension 2	Additional PNT → SRL and PNT → CT paths	Post hoc exploratory
GGC-adjusted model	Adjustment for GenAI guidance clarity	Robustness
Covariate-adjusted model	Adjustment for gender, discipline, year level, and institution	Robustness
DWLS model	Ordinal-item estimator	Sensitivity
Reverse focal model	LOU → PNT replacing PNT → LOU	Directionality sensitivity
Institution analyses	Leave-one-institution-out and institution-specific models	Sensitivity
Split-sample analysis	Calibration EFA and held-out CFA	Internal replication

The study was not preregistered. “A priori theoretical specification” refers to the six structural paths specified before estimation of the post-hoc exploratory extensions and should not be interpreted as preregistered confirmatory analysis. Robustness, sensitivity, and internal-replication analyses were not treated as additional confirmatory hypothesis tests.

## Results

### Preliminary analysis

Descriptive statistics and bivariate correlations among the study constructs are presented in Table 3. Overall responses reflected moderately positive levels across the measured constructs. LOU had the highest mean score (M = 3.903, SD = 0.644), followed by SRL (M = 3.884, SD = 0.640) and PNT (M = 3.860, SD = 0.682). ACS (M = 3.781, SD = 0.695) and CT (M = 3.737, SD = 0.722) showed similar levels, whereas GGC had the lowest mean (M = 3.655, SD = 0.746). The lower GGC mean indicates comparatively less agreement that institutional and course-level GenAI guidance was clear and consistent and should not be interpreted as evidence that such guidance was uniformly absent. Construct-level skewness values ranged from −0.391 to −0.263 and kurtosis values from −0.474 to −0.268, indicating no substantial departures from univariate normality.

**Table 3:** Descriptive statistics and bivariate correlations among student constructs (*N* = 1,004).

Construct	M	SD	1	2	3	4	5	6
1. PNT	3.860	0.682	—					
2. LOU	3.903	0.644	0.453	—				
3. ACS	3.781	0.695	0.404	0.406	—			
4. SRL	3.884	0.640	0.327	0.398	0.395	—		
5. CT	3.737	0.722	0.297	0.348	0.329	0.446	—	
6. GGC	3.655	0.746	0.295	0.316	0.297	0.362	0.424	—

PNT = perceived teacher-referenced GenAI normative tension; LOU = self-reported learning-oriented GenAI use; ACS = combined autonomy- and competence-related academic experience; SRL = self-regulated learning; CT = self-reported critical-evaluation tendencies; GGC = GenAI guidance clarity. Correlations are Pearson correlations among observed five-item composite scores. All correlations were statistically significant at *p* < 0.001.

The unmatched faculty sample was used only to provide aggregate contextual information and not to validate student-level PNT. Student and faculty means were similar for perceived usefulness, learning facilitation, and risk perception. Faculty members reported greater ethical-boundary strictness than students, Welch’s t(190.15) = −4.01, *p* < 0.001, Cohen’s d = −0.36; this was the only group difference that remained significant under the Bonferroni-adjusted criterion. Because the student and faculty samples were unmatched and did not complete parallel PNT measures, this aggregate difference should not be interpreted as evidence of classroom-level teacher–student discrepancy or as multi-source validation of PNT. Full comparisons are reported in Supplementary Table S5.

### Measurement model assessment

The hypothesised five-factor measurement model showed unusually close approximate fit, χ^2^(265) = 252.76, *p* = 0.695, CFI = 1.000, TLI = 1.000, RMSEA = 0.000, 90% CI [0.000, 0.010], and SRMR = 0.020. Given this uncommon degree of fit, the model was re-estimated from the original item-level data and additional parameter, residual, and modification-index diagnostics were examined. Standardised loadings ranged from 0.697 to 0.772 (SEs = 0.016–0.020), with standardised error variances of 0.404–0.515. The mean absolute residual correlation was 0.016, no residual correlation exceeded |0.10|, and the largest absolute residual was 0.066. The largest modification index was 9.26 and concerned a potential cross-loading of LOU2 on PNT; no post-hoc cross-loadings or correlated residuals were introduced. Full item-level estimates are reported in Supplementary Tables S9–S11. Although these diagnostics did not indicate substantial localised misspecification and the close fit was reproduced in the held-out validation subsample, the unusually close fit should not be treated as definitive evidence of measurement validity. Homogeneous item wording, shared-method characteristics, or other sample-specific covariance features remain possible contributors, and independent external replication is required. Measurement-quality indices are summarised in Table 4.

**Table 4:** Measurement model results.

Construct	Loading range	α	CR	AVE	√AVE	Max. HTMT
PNT	0.720–0.766	0.864	0.865	0.561	0.749	0.534
LOU	0.697–0.728	0.835	0.835	0.503	0.709	0.534
ACS	0.716–0.772	0.859	0.859	0.550	0.741	0.479
SRL	0.704–0.731	0.844	0.844	0.520	0.721	0.524
CT	0.720–0.750	0.858	0.858	0.547	0.740	0.524

α = Cronbach’s alpha; CR = composite reliability; AVE = average variance extracted; HTMT = heterotrait–monotrait ratio. All standardised loadings were statistically significant (*p* < 0.001).

Discriminant validity was evaluated using latent-factor correlations, HTMT values, square-root-of-AVE comparisons, and alternative measurement-model tests (Henseler et al., 2015). Latent correlations ranged from 0.345 to 0.534, and all HTMT values were below 0.85, with a maximum of 0.534. The complete latent-correlation and HTMT matrices are reported in Supplementary Table S10. The square root of AVE for each construct exceeded its correlations with the remaining constructs.

The five PNT indicators loaded coherently on the proposed factor (0.720–0.766). Because the items represent potentially distinguishable aspects of teacher-referenced normative difficulty, the one-factor representation was compared with alternative two-factor specifications. The unconstrained two-factor model fit significantly better than the one-factor model, Δχ^2^(1) = 8.37, *p* = 0.004, but the estimated correlation between the two factors was 0.949, indicating very limited empirical separation. PNT was therefore retained as a single factor for the present analysis on grounds of parsimony and the high interfactor correlation, rather than because the one-factor representation was uniquely supported. Because no dimensionality criterion was preregistered, no retrospective threshold is presented as an *a priori* decision rule. Future preregistered validation should specify in advance the empirical conditions under which a multidimensional representation would be preferred. Accordingly, the present one-factor representation should be treated as a parsimonious, sample-specific summary rather than as evidence of established unidimensionality. Full comparisons are reported in Supplementary Table S12.

The combined ACS specification was also examined against a constrained two-factor model separating autonomy and competence. The one-factor model showed close fit, χ^2^(5) = 5.41, *p* = 0.368, CFI = 1.000, TLI = 1.000, RMSEA = 0.009, and SRMR = 0.009. Although the constrained two-factor model also showed superficially close global fit, the estimated latent correlation between autonomy and competence was 1.029, yielding an inadmissible solution. The five items were therefore retained pragmatically as a combined ACS factor because the available item set did not support an admissible empirical separation of autonomy and competence. This decision does not imply that autonomy and competence are theoretically interchangeable. Full results are reported in Supplementary Table S3.

### Internal split-sample measurement analysis

As an additional internal measurement check, the final sample was randomly divided into calibration and validation subsamples of 502 respondents each. Parallel analysis in the calibration subsample retained five factors. Principal-axis exploratory factor analysis with oblimin rotation produced a pattern in which the indicators loaded primarily on their intended factors, with no substantively large cross-loadings. The resulting five-factor representation was then evaluated in the held-out validation subsample. The validation CFA showed excellent fit, χ^2^(265) = 269.02, *p* = 0.420, CFI = 0.999, TLI = 0.999, RMSEA = 0.006, and SRMR = 0.030. Standardised loadings remained consistent with the intended five-factor structure. These findings provide an internal replication of the measurement representation within the present dataset but should not be interpreted as independent external validation because both subsamples were drawn from the same institutions, sampling frame, and data-collection period. Parallel-analysis results, exploratory factor loadings, and validation-subsample CFA estimates are reported in Supplementary Tables S13a–c.

The hypothesised five-factor model fit substantially better than the alternative measurement specifications. Combining LOU and SRL produced markedly poorer fit, χ^2^ = 1367.31, CFI = 0.897, RMSEA = 0.064, whereas combining LOU, SRL, and CT resulted in further deterioration, χ^2^ = 2439.30, CFI = 0.798, RMSEA = 0.089. The single-factor model fit poorly, χ^2^ = 4869.15, CFI = 0.571, RMSEA = 0.129, indicating that the focal constructs did not collapse into a single general self-reported learning tendency. Complete fit indices and χ^2^ difference tests are reported in Supplementary Table S2.

An extended six-factor model including GGC also showed very close approximate fit, *x*^2^(390) = 386.05, *p* = 0.547, CFI = 1.000, TLI = 1.000, RMSEA = 0.000, 90% CI [0.000, 0.011], and SRMR = 0.021. The six-factor representation was consistent with treating PNT and GGC as separate latent factors within the present sample, although this should not be interpreted as evidence of complete conceptual independence between the two adjacent constructs. The model was estimated without item parcels or correlated residuals. Additional measurement-quality information is reported in Supplementary Table S11.

### Primary structural results

The theoretically specified six-path model showed generally acceptable global fit, χ^2^(269) = 400.03, *p* < 0.001, CFI = 0.988, TLI = 0.986, RMSEA = 0.022, and SRMR = 0.055, although the SRMR exceeded the more stringent 0.05 benchmark. The SRMR nevertheless remained below the 0.08 criterion commonly used to indicate acceptable residual fit (Hu and Bentler, 1999). Relative to the unusually close measurement-model fit, this higher SRMR nevertheless indicated that the theory-constrained structural specification did not fully reproduce all observed covariances. All six specified coefficients were positive and statistically significant and were consistent with H1–H6 within the theory-constrained model. The model is therefore interpreted as a theoretically focal but structurally incomplete specification rather than as an exhaustive representation of the covariance structure. Figure 2 displays the standardised coefficients and explained variance, while complete estimates are reported in Table 5.

**Figure 2:**
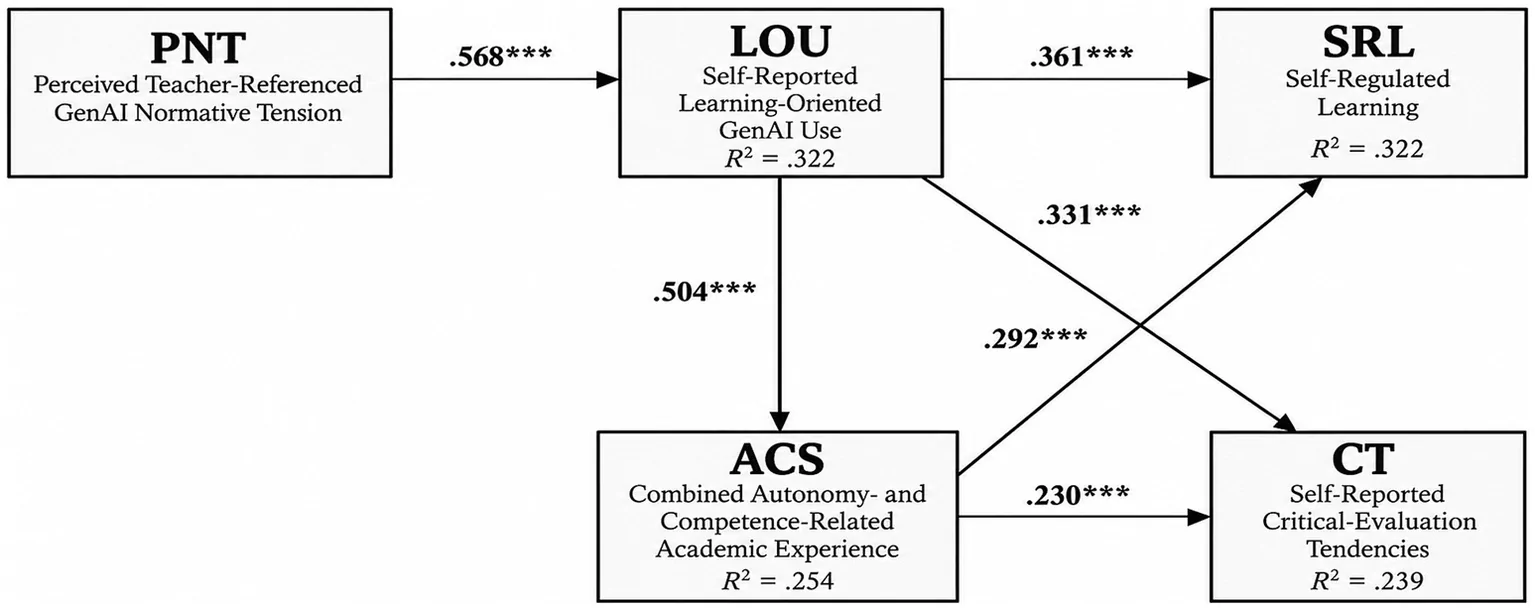
Standardised estimates for the hypothesised structural model. Values are standardised coefficients from the theoretically specified six-path model. R^2^ values are shown for endogenous constructs, and all displayed coefficients were statistically significant at *p* < 0.001. Directional arrows represent theory-constrained model specification and do not establish temporal or causal effects. Exploratory extensions reported separately showed additional direct associations from PNT and indicated that the displayed model does not exhaust the observed covariance structure.

**Table 5:** Hypothesised structural model results.

Path	β	Status
PNT → LOU	0.568	Consistent with H1
LOU → ACS	0.504	Consistent with H2
LOU → SRL	0.361	Consistent with H3
LOU → CT	0.331	Consistent with H4
ACS → SRL	0.292	Consistent with H5
ACS → CT	0.230	Consistent with H6

β = standardised path coefficient. All coefficients were positive and statistically significant at *p* < 0.001 and were consistent with H1–H6. The directional specification does not establish temporal or causal precedence. Model fit: χ^2^(269) = 400.03, CFI = 0.988, TLI = 0.986, RMSEA = 0.022, SRMR = 0.055. Standard errors for the primary structural coefficients are reported in Supplementary Table S14c.

The six-path model explained 32.2% of the variance in LOU, 25.4% in ACS, 32.2% in SRL, and 23.9% in CT. The coefficients of determination for all endogenous constructs are summarised in Table 6.

**Table 6:** Explained variance in the hypothesised model.

Endogenous construct	R^2^
LOU	0.322
ACS	0.254
SRL	0.322
CT	0.239

R^2^ = proportion of variance explained by the theoretically specified six-path model.

### Algebraic indirect associations implied by the specified model

The theoretically specified model algebraically implied positive indirect associations from PNT to ACS, SRL, and CT. Bootstrap confidence intervals for these model-implied products excluded zero. These quantities are reported for descriptive completeness only. Because temporal ordering was not identified, the reverse focal specification was observationally equivalent, and exploratory direct paths indicated that the six-path structure was not exhaustive, the estimates are not interpreted as mediation, transmission processes, or evidence that LOU carries an effect of PNT to the broader learning constructs. Complete estimates are reported in Supplementary Table S4.

### Exploratory structural extensions

Exploratory models examined whether the six-path focal model omitted additional direct associations between PNT and the broader learning constructs. Adding a direct path from PNT to ACS substantially improved model fit, Δχ^2^(1) = 52.87, *p* < 0.001, and reduced SRMR from 0.055 to 0.045. In this model, PNT remained positively associated with LOU, β = 0.544, *p* < 0.001, while both LOU and PNT were positively associated with ACS, β = 0.311 and β = 0.304, respectively, both *p* < 0.001. The reduction in the LOU → ACS coefficient from 0.504 to 0.311 indicates that the six-path specification did not fully represent covariance involving ACS.

Adding direct paths from PNT to SRL and CT produced a further but smaller improvement, Δχ^2^(2) = 9.05, *p* = 0.011, with SRMR decreasing to 0.044. In the fully expanded model, the PNT → SRL and PNT → CT associations were small, β = 0.092, *p* = 0.030, and β = 0.103, *p* = 0.018, respectively. PNT remained positively associated with LOU, β = 0.535, *p* < 0.001, and PNT → ACS remained positive, β = 0.297, *p* < 0.001. These findings further indicate that the six-path model is theoretically focal but structurally incomplete and does not exhaust the observed covariance structure. Because the additional direct paths were examined after estimation of the hypothesised model and were not part of the *a priori* theoretical specification, they are retained as exploratory findings rather than incorporated into the primary model. Complete fit indices and structural coefficients are reported in Supplementary Tables S14a,b.

### Robustness of the focal association

Adjustment for GenAI guidance clarity did not eliminate the focal association. The six-factor robustness model fit the data well, χ^2^(396) = 625.61, CFI = 0.982, TLI = 0.981, and RMSEA = 0.024. PNT remained positively associated with LOU, β = 0.478, *p* < 0.001, while GGC was independently associated with LOU, β = 0.243, *p* < 0.001, and ACS, β = 0.196, *p* < 0.001. The explained variance in LOU increased from 32.2 to 36.9%. Thus, PNT showed an incremental association with LOU beyond perceived guidance clarity, although its coefficient was attenuated from β = 0.568 to β = 0.478. This attenuation indicates overlap between PNT and GGC but is interpreted as evidence of incremental association rather than as a standalone test of discriminant validity. Complete results are reported in Supplementary Table S6.

The focal coefficient was also stable after adjustment for gender, discipline, year level, and institution. In the covariate-adjusted model, PNT → LOU was β = 0.570, *p* < 0.001, compared with β = 0.568 in the unadjusted model. The adjusted model fit well, χ^2^(389) = 494.72, CFI = 0.990, TLI = 0.989, and RMSEA = 0.016. Humanities and social-science enrolment showed a small negative association with LOU, β = −0.064, *p* = 0.034; the remaining demographic and institutional covariates did not materially alter the focal result.

Leave-one-institution-out analyses showed that the focal association remained positive and statistically significant after each institution was excluded. The standardised PNT–LOU coefficient was 0.576 after excluding Institution A, 0.550 after excluding Institution B, and 0.581 after excluding Institution C. Institution-specific models produced similarly positive coefficients for Institution A (β = 0.552), Institution B (β = 0.606), and Institution C (β = 0.555). These findings indicate that the focal association was not attributable exclusively to one institution, although the analyses do not eliminate all possible dependence within shared institutional or instructional contexts. Full results are reported in Supplementary Table S8.

The ordinal-item DWLS sensitivity model also showed acceptable fit, χ^2^(269) = 568.72, CFI = 0.989, TLI = 0.987, and RMSEA = 0.033, and all six specified associations remained statistically significant. Coefficient magnitudes were more sensitive for the ACS-related paths, particularly ACS → SRL and ACS → CT, indicating that estimator choice affected their magnitude more than their statistical significance. Complete ML and DWLS estimates are reported in Supplementary Table S7.

### Directionality and observational equivalence

A model reversing the focal association to LOU → PNT was observationally equivalent to the hypothesised specification and therefore produced the same fit, χ^2^(269) = 400.03, CFI = 0.988, TLI = 0.986, RMSEA = 0.022, and SRMR = 0.055, together with equivalent information criteria. This result is not interpreted as support for the reverse direction. Instead, it demonstrates that the cross-sectional covariance structure cannot distinguish whether PNT precedes LOU or whether LOU precedes PNT. The focal result is therefore interpreted as a positive association without identified temporal direction; the same limitation applies to the remaining structural paths.

## Discussion

The central finding was a positive association between perceived teacher-referenced normative tension and self-reported learning-oriented GenAI use. This result is consistent with emerging evidence that students’ AI-related academic conduct is shaped by perceived ethical and instructor-referenced norms rather than by institutional policy awareness alone (Lund et al., 2025; Huang et al., 2025). The present findings extend this work by showing that difficulty positioning one’s GenAI practices relative to teacher expectations can coexist with stronger learning-oriented self-reports. Importantly, the association remained positive after adjustment for perceived guidance clarity, although its attenuation from β = 0.568 to β = 0.478 indicates meaningful overlap between normative-positioning difficulty and guidance clarity. One plausible interpretation is that students who continue using GenAI under unsettled expectations place greater emphasis on practices that preserve visible cognitive involvement and are easier to justify academically. An alternative is that students who already use GenAI reflectively become more attentive to teacher-referenced normative boundaries. The cross-sectional design cannot distinguish these interpretations, but the pattern identifies teacher-referenced expectations as a relevant proximal context for how students describe their GenAI use.

Students who described their GenAI use in more learning-oriented terms also reported stronger combined autonomy- and competence-related academic experience, self-regulated learning, and critical-evaluation tendencies. This pattern is consistent with the view that GenAI use varies in cognitive quality rather than constituting a unitary academic behaviour. At the same time, prior evidence that AI use can foster over-reliance or reduce metacognitive processing cautions against equating self-reported learning orientation with objectively improved learning (Zhai et al., 2024; Bauer et al., 2025). The observed associations may therefore reflect substantively connected learning orientations, but common-method covariance, general academic engagement, and academically desirable self-presentation remain plausible contributors. CT had the lowest explained variance (R^2^ = 0.239), further suggesting that self-reported LOU and ACS alone are insufficient to account for higher-order evaluation without considering instructional scaffolding, assessment design, disciplinary epistemic practices, and AI-literacy training (Ng et al., 2024; Xia et al., 2023).

### Implications of the exploratory PNT–ACS Association

The exploratory PNT–ACS association materially qualifies the interpretation of the six-path model. Adding this direct path improved model fit, and the LOU → ACS coefficient decreased from 0.504 to 0.311 while PNT showed an independent association with ACS of β = 0.304. This pattern indicates that covariance involving ACS was not adequately represented by a sequence running only from PNT through LOU. PNT and ACS may share antecedents such as academic self-efficacy, agency, normative attentiveness, or general positive response tendencies, but these possibilities were not measured. The result therefore argues against interpreting the six-path specification as fully accounting for the association between normative-positioning difficulty and autonomy- and competence-related academic experience and supports retaining ACS as a secondary academic correlate rather than evidence of a Self-Determination Theory mechanism. The primary six-path model is consequently better understood as a focal nomological specification centred on PNT–LOU than as a complete explanatory process.

### Theoretical contributions

The primary contribution is a bounded conceptual distinction between students’ perceived difficulty positioning their GenAI practices relative to teacher-referenced expectations and their self-reported orientation toward learning-oriented GenAI use. The positive association indicates that these experiences were related but non-equivalent in the present sample and need not be conceptualised as opposite ends of a single continuum. At the same time, the measurement evidence does not establish PNT as a mature unidimensional scale: the proposed subdimensions showed limited empirical separation, and independent construct-development work is needed to determine whether disagreement, alignment uncertainty, unclear expectations, and cross-teacher inconsistency become reproducibly distinguishable under other governance conditions.

A second contribution concerns construct differentiation. The measurement comparisons indicate that self-reported GenAI-specific learning orientation is distinguishable from domain-general self-regulation. LOU captures reported technology-specific practices such as concept clarification, feedback interpretation, and output evaluation, whereas SRL concerns broader processes of planning, monitoring, and adjustment across learning tasks (Panadero, 2017; Schunk and Zimmerman, 2011). Self-reported learning-oriented GenAI use should therefore not be treated simply as a technological expression of general SRL. At the same time, both measures remain self-reports and may share academically desirable response tendencies.

An additional pattern concerned the association between LOU and broader autonomy- and competence-related academic experience. Because the ACS measure did not empirically distinguish autonomy from competence and was not specific to GenAI use, this result does not constitute a direct test of Self-Determination Theory or evidence of a psychological-need mechanism (Ryan and Deci, 2020). It instead indicates that students reporting more learning-oriented GenAI use also reported more positive autonomy- and competence-related academic experiences. The temporal direction and explanatory significance of this association remain unresolved.

### Practical implications

The practical implications follow from both the present findings and prior evidence that instructor-referenced norms may be especially proximal to students’ AI-related academic conduct (Huang et al., 2025). The positive PNT–LOU association should not be interpreted as a reason to preserve ambiguous or inconsistent GenAI expectations. Rather, the coexistence of normative tension and learning-oriented self-reports suggests that institution-level policy may be insufficient unless broad principles are translated into clear course- and assignment-specific expectations. Clear acceptable-use guidance therefore remains necessary on ethical, procedural, and pedagogical grounds (Mumtaz et al., 2025), particularly because GGC received the lowest mean among the measured constructs. At the institutional level, universities should distinguish permitted, restricted, and prohibited forms of GenAI use across different types of academic tasks and provide consistent expectations concerning disclosure, citation, and responsibility for generated content.

At the course level, teachers should state whether GenAI use is permitted for each major assignment, identify acceptable functions such as brainstorming, concept clarification, language support, or feedback, and provide examples distinguishing learning support from task substitution. Where GenAI use is permitted, students may be asked to document prompts, evaluate the accuracy of generated content, explain how outputs were revised, and identify the contribution of their own reasoning. Such process-oriented requirements may make expectations more transparent while preserving visible student cognitive involvement.

Faculty development should focus on translating broad institutional principles into assignment-specific expectations, including whether GenAI is permitted, which functions are acceptable, how use should be disclosed, and what evidence of independent student reasoning is required (Chiu, 2024; Faraon et al., 2025). The policy implication is therefore not to create productive tension, but to reduce avoidable ambiguity while making academically responsible forms of GenAI engagement explicit, teachable, and assessable. The present findings do not demonstrate that these practices would cause improvements in learning outcomes.

### Limitations and future research

Several limitations should be acknowledged. The cross-sectional design prevents causal and temporal inference, and the observationally equivalent reverse specification could not determine whether PNT preceded LOU or vice versa. All focal constructs were measured through same-source self-reports at a single time point. Although the hypothesised measurement model outperformed a single-factor alternative and was reproduced in a held-out subsample, these findings do not eliminate common-method inflation, socially desirable responding, or general positive response tendencies. This concern is particularly relevant to LOU because its items describe academically responsible and cognitively active GenAI use; students more attentive to teacher-referenced norms may also have been more likely to describe their own use in pedagogically legitimate terms. Future research should therefore incorporate longitudinal, multi-source, behavioural, and experimental evidence.

The sampling and measurement design also limits the scope of the conclusions. Participants were drawn from three Chinese public universities and were mainly sophomore and junior students, with humanities and social science students overrepresented. Transferability to other institutional, disciplinary, year-level, and national contexts therefore remains uncertain, and measurement invariance and structural equivalence across groups were not tested. Course- and teacher-level identifiers were unavailable, the teacher sample was not matched to individual students or classrooms, and the inclusion of only three institutions prevented reliable multilevel modelling or institution-level cluster-robust inference. Although leave-one-institution-out and institution-specific analyses indicated that the focal association was not driven by a single university, these checks cannot fully address dependence within shared instructional contexts.

The measures also require further validation. Although the five-factor structure was reproduced in a held-out subsample and the PNT indicators were compared under alternative dimensional specifications, these analyses provide only internal evidence from a single dataset. They do not establish external replicability, cross-contextual measurement invariance, or the stability of the factor structure across institutions, countries, disciplines, or forms of GenAI governance. PNT was assessed using a context-specific five-item adaptation rather than an established standalone scale. Although PNT and GGC could be modelled as separate latent factors within the present sample, some item content overlapped around perceived clarity and consistency, and this separation should not be interpreted as complete conceptual independence. Future construct-development work should sharpen the boundary between perceived properties of communicated guidance and students’ difficulty positioning their own practices relative to teacher-referenced expectations. LOU, SRL, and CT were operationalised using study-specific self-report items, and conceptual overlap between LOU and broader self-regulatory or academically desirable response tendencies cannot be fully excluded. ACS was measured as a general academic experience rather than a GenAI-specific state, and the five-item measure did not empirically distinguish autonomy from competence.

The study also lacked detailed information about duration of GenAI use, tool type, task context, and use intensity. Future research should therefore validate the focal measures in independent samples, test adjacent and multidimensional specifications, use matched and multilevel designs, and incorporate behavioural traces or performance-based measures.

## Conclusion

This study identified a positive association between perceived teacher-referenced GenAI normative tension and self-reported learning-oriented GenAI use among Chinese university students, including after adjustment for perceived guidance clarity and across multiple sensitivity analyses. The findings show that normative-positioning difficulty and learning-oriented self-reports can coexist, while the exploratory PNT–ACS association indicates that the six-path model does not exhaust the observed covariance structure. Because the evidence is cross-sectional and self-reported, temporal direction and behavioural consequences remain unresolved. Independent longitudinal, multi-source, and behavioural research is needed to determine how teacher-referenced GenAI norms relate to students’ learning practices over time.

## Data Availability

The raw data supporting the conclusions of this article will be made available by the authors, without undue reservation.
