# Supplementary material for: Self-reported learning-oriented GenAI use under perceived teacher-referenced normative tension in Chinese higher education
Source: Front Psychol. 2026 Oct 2;17:1969743. doi: 10.3389/fpsyg.2026.1969743 (PMC13672657; doi:10.3389/fpsyg.2026.1969743)
Supplement: Supplementary file 1 [file Supplementary_file_1.DOCX]

**Supplementary Materials**

Self-Reported Learning-Oriented GenAI Use Under Perceived Teacher-Referenced Normative Tension in Chinese Higher Education

This document provides supplementary measurement and analytical information referenced in the manuscript. All student analyses used the final valid sample of 1,004 respondents. PNT is treated as an initial, context-specific operational representation; LOU and CT are self-reported orientations or tendencies rather than observed behaviour or performance. ACS represents combined autonomy- and competence-related academic experience. The unmatched teacher sample (N = 148) was used only for supplementary group-level descriptive context and did not provide multi-source validation of PNT. Covariance-based structural equation modelling used maximum-likelihood estimation unless otherwise stated. Directional paths are theory-based specifications and do not establish temporal or causal precedence. H1 represents the focal theoretical association. The remaining structural paths provide nomological positioning for LOU, and the exploratory extensions assess whether the six-path specification exhausts the observed covariance structure.

**S1. Full Item Wording and Original Questionnaire Codes**

Table S1 reports the complete English wording and original questionnaire codes for the six student measures included in the focal measurement model or the GenAI guidance clarity robustness extension. The PNT items retained their original PTM1–PTM5 codes, and the ACS items retained their original NS1–NS5 codes. The wording shown in Table S1 reflects the final post-pilot questionnaire and was unchanged after main data collection.

**Table S1**

*Full Item Wording and Original Questionnaire Codes for Student Measures*

| **Manuscript construct** | **Original code** | **Item wording** | **Source/adaptation** |
| --- | --- | --- | --- |
| PNT | PTM1 | My teachers and I seem to hold different views about appropriate GenAI use in academic work. | Contextually adapted from Rizzo et al. (1970) and Sawyer (1992) |
| PNT | PTM2 | I am often unsure whether my use of GenAI aligns with my teachers' expectations. | Same adaptation |
| PNT | PTM3 | There is a gap between how students use GenAI and how teachers think it should be used. | Same adaptation |
| PNT | PTM4 | My teachers' expectations about GenAI use are not always clear to students. | Same adaptation |
| PNT | PTM5 | Different teachers seem to apply different standards for acceptable GenAI use. | Same adaptation |
| LOU | LOU1 | I use GenAI to deepen my understanding of complex concepts rather than simply obtain answers. | Developed for the present study |
| LOU | LOU2 | When using GenAI, I critically evaluate its responses rather than accept them directly. | Developed for the present study |
| LOU | LOU3 | I use GenAI to broaden my understanding of course-related topics through different perspectives. | Developed for the present study |
| LOU | LOU4 | I integrate GenAI feedback with my own reasoning. | Developed for the present study |
| LOU | LOU5 | I use GenAI to identify weaknesses in my understanding or learning process. | Developed for the present study |
| ACS | NS1 | In my learning, I feel I can make my own choices. | Adapted from Chen et al. (2015) |
| ACS | NS2 | I feel that I can study in ways that suit my interests and goals. | Adapted from Chen et al. (2015) |
| ACS | NS3 | I feel a sense of personal control over my learning. | Adapted from Chen et al. (2015) |
| ACS | NS4 | In my learning, I feel capable of handling difficult tasks. | Adapted from Chen et al. (2015) |
| ACS | NS5 | I feel confident in solving academic problems. | Adapted from Chen et al. (2015) |
| SRL | SRL1 | I set clear learning goals before starting academic tasks. | Developed for the present study with reference to Schunk and Zimmerman (2011) |
| SRL | SRL2 | I plan how to complete my learning tasks before I begin. | Same development basis |
| SRL | SRL3 | I monitor whether I understand the material while studying. | Same development basis |
| SRL | SRL4 | I adjust my learning strategies when I encounter difficulties. | Same development basis |
| SRL | SRL5 | After completing a task, I reflect on how I could improve my learning. | Same development basis |
| CT | CT1 | I usually question whether information is reliable before accepting it. | Developed for the present study as a self-report measure of critical-evaluation tendencies |
| CT | CT2 | I try to examine different viewpoints before forming a conclusion. | Same development basis |
| CT | CT3 | I look for evidence to support academic claims. | Same development basis |
| CT | CT4 | I can identify weaknesses in arguments or explanations. | Same development basis |
| CT | CT5 | I reflect on my own assumptions when solving academic problems. | Same development basis |
| GGC | GGC1 | My institution provides clear guidance on acceptable GenAI use. | Context-specific measure |
| GGC | GGC2 | My teachers explicitly tell students which assignments allow GenAI use and which do not. | Context-specific measure |
| GGC | GGC3 | I know exactly what counts as inappropriate GenAI use according to my institution. | Context-specific measure |
| GGC | GGC4 | The expectations for disclosing GenAI use are clear in my courses. | Context-specific measure |
| GGC | GGC5 | My courses provide consistent rules about acceptable GenAI use. | Context-specific measure |

*Note. All items were rated on a five-point Likert scale ranging from 1 (strongly disagree) to 5 (strongly agree). PNT is a provisional, context-specific latent summary of related teacher-referenced normative experiences. Its indicators cover perceived disagreement, uncertainty about alignment, unclear expectations, and inconsistency across teachers. The present one-factor specification does not establish that these experiences are interchangeable or universally unidimensional. LOU represents self-reported learning-oriented GenAI use, CT represents self-reported critical-evaluation tendencies, and ACS represents combined autonomy- and competence-related academic experience. The ACS specification does not imply that autonomy and competence are theoretically identical.*

**S2. Measurement-Model Comparisons**

The hypothesised five-factor model was compared with specifications combining conceptually adjacent learning constructs and with a single-factor model. A six-factor extension including GGC was also estimated. The five-factor model fit substantially better than the alternative collapsed-factor specifications.

**Table S2**

*Comparison of the Hypothesised Measurement Model with Alternative Specifications*

| **Model** | **χ²** | **df** | **CFI** | **TLI** | **RMSEA** | **SRMR** | **Δχ²** | **Δdf** | **p** |
| --- | --- | --- | --- | --- | --- | --- | --- | --- | --- |
| Five-factor | 252.756 | 265 | 1.000 | 1.000 | .000 | .020 | — | — | — |
| LOU and SRL combined | 1367.315 | 269 | .897 | .886 | .064 | .057 | 1114.558 | 4 | < .001 |
| LOU, SRL, and CT combined | 2439.297 | 272 | .798 | .777 | .089 | .080 | 2186.541 | 7 | < .001 |
| Single-factor | 4869.148 | 275 | .571 | .532 | .129 | .111 | 4616.392 | 10 | < .001 |
| Six-factor including GGC | 386.046 | 390 | 1.000 | 1.000 | .000 | .021 | — | — | — |

*Note. Difference tests compare each collapsed-factor model with the hypothesised five-factor model. Values of CFI and TLI exceeding 1.000 are conventionally reported as 1.000.*

**S3. Autonomy and Competence Measurement Comparison**

Because autonomy and competence are theoretically distinct, a constrained two-factor specification separating NS1–NS3 from NS4–NS5 was compared with the combined one-factor ACS model. The constrained two-factor model produced a latent autonomy–competence correlation of 1.029 and was therefore inadmissible. This result indicates that autonomy and competence could not be empirically separated using the present abbreviated five-item measure. It does not establish that the two needs are theoretically identical. The combined factor was retained only as a pragmatic, sample-specific representation for the present analysis.

**Table S3**
*One-Factor and Constrained Two-Factor Specifications for ACS*

| **Model** | **χ²** | **df** | **CFI** | **TLI** | **RMSEA** | **SRMR** | **AIC** | **BIC** |
| --- | --- | --- | --- | --- | --- | --- | --- | --- |
| One-factor ACS | 5.411 | 5 | 1.000 | 1.000 | .009 | .009 | 19.989 | 69.107 |
| Two-factor autonomy + competence | 2.958 | 4 | 1.000 | 1.000 | .000 | .007 | 21.994 | 76.023 |

*Note. The constrained two-factor model was not retained because the estimated latent correlation between autonomy and competence exceeded unity (r = 1.029), indicating an inadmissible solution. The one-factor representation is retained pragmatically as a sample-specific measure of combined autonomy- and competence-related academic experience because the present abbreviated item set did not support an admissible empirical separation of the two dimensions. It should not be interpreted as evidence that autonomy and competence are theoretically identical or as a formal test of the multidimensional need structure proposed by Self-Determination Theory.*

**S4. Algebraic Indirect Associations Implied by the Specified Model**

Standardised indirect products were estimated using 5,000 bootstrap resamples. These quantities are reported for descriptive completeness as algebraic implications of the specified covariance model. Because temporal ordering was not identified, the reverse focal specification was observationally equivalent, and exploratory direct paths indicated that the six-path structure was not exhaustive, the estimates are not interpreted as mediation, transmission processes, or evidence that LOU carries an effect of PNT to the broader learning constructs.

**Table S4**

*Standardised Model-Implied Indirect Associations*

| **Model-implied product** | **β** | **SE** | **95% CI** |
| --- | --- | --- | --- |
| PNT → LOU → ACS | .286 | .026 | [.236, .338] |
| PNT → LOU → SRL | .205 | .027 | [.155, .259] |
| PNT → LOU → CT | .188 | .027 | [.138, .243] |
| PNT → LOU → ACS → SRL | .083 | .014 | [.057, .112] |
| PNT → LOU → ACS → CT | .066 | .014 | [.038, .094] |
| LOU → ACS → SRL (context) | .147 | .023 | [.104, .193] |
| LOU → ACS → CT (context) | .116 | .024 | [.070, .162] |

*Note. All confidence intervals excluded zero. Total model-implied indirect association of PNT: SRL β = .289; CT β = .254. These quantities are algebraic products within the specified covariance model and do not establish temporal mediation, causal transmission, or an exhaustive structural process.*

**S5. Aggregate Teacher–Student Comparison**

The unmatched teacher sample is reported only as supplementary descriptive context and was not part of the focal student-level measurement or structural analyses. Teacher responses were not linked to individual students, courses, classrooms, or instructors and did not assess teacher-side equivalents of the student PNT items. Accordingly, these data were not used to compute, validate, or externally corroborate student-level PNT. Four shared five-item GenAI-related dimensions were compared descriptively using Welch’s independent-samples t-tests. Under a Bonferroni-adjusted criterion of α = .0125, only the ethical-boundary strictness difference was statistically significant.

**Table S5**

*Aggregate Comparison Between Unmatched Student and Teacher Samples*

| **Dimension** | **Student M (SD)** | **Teacher M (SD)** | **Welch t** | **df** | **p** | **Cohen’s d** | **Student α** | **Teacher α** |
| --- | --- | --- | --- | --- | --- | --- | --- | --- |
| Perceived usefulness | 4.149 (.582) | 4.139 (.620) | .181 | 187.124 | .856 | .017 | .866 | .907 |
| Ethical-boundary strictness | 3.942 (.605) | 4.161 (.623) | -4.008 | 190.152 | < .001 | −.361 | .826 | .893 |
| Learning facilitation | 4.037 (.585) | 4.023 (.660) | .246 | 182.628 | .806 | .024 | .832 | .899 |
| Risk perception | 4.001 (.608) | 4.041 (.570) | −.782 | 199.802 | .435 | −.066 | .862 | .831 |

*Note. Student N = 1,004; teacher N = 148. The comparisons indicate group-level tendencies only, do not establish teacher–student disagreement within particular classrooms, and do not constitute multi-source validation of PNT.*

**S6. Robustness of the Focal PNT–LOU Association**

The focal association remained positive after adjustment for GGC, demographic and institutional covariates, and use of an ordinal estimator. The reverse focal model produced equivalent global fit, illustrating that temporal direction could not be identified.

**Table S6**

*Focal Association Across Model Specifications*

| **Specification** | **Focal standardised coefficient** | **Model fit** |
| --- | --- | --- |
| Baseline six-path (ML) | .568 | χ²(269) = 400.03; CFI = .988; TLI = .986; RMSEA = .022 |
| Adjusted for GGC | .478 | χ²(396) = 625.61; CFI = .982; TLI = .981; RMSEA = .024 |
| Adjusted for demographics and institution | .570 | χ²(389) = 494.72; CFI = .990; TLI = .989; RMSEA = .016 |
| Ordinal estimator (DWLS) | .647 | χ²(269) = 568.72; CFI = .989; TLI = .987; RMSEA = .033 |
| Reverse focal (LOU → PNT) | Reverse coefficient not interpreted | χ²(269) = 400.03; CFI = .988; TLI = .986; RMSEA = .022 |

*Note. The reverse focal specification reproduced the same global fit and covariance structure. This equivalence does not identify temporal direction or imply equivalent substantive interpretations of the forward and reverse specifications.*

**S7. Structural Coefficients Across Estimators**

**Table S7**

*Standardised Structural Coefficients Under ML and DWLS*

| **Path** | **ML β** | **DWLS β** |
| --- | --- | --- |
| PNT → LOU (H1) | .568 | .647 |
| LOU → ACS (H2) | .504 | .595 |
| LOU → SRL (H3) | .361 | .520 |
| LOU → CT (H4) | .331 | .484 |
| ACS → SRL (H5) | .292 | .175 |
| ACS → CT (H6) | .230 | .125 |

*Note. All coefficients were statistically significant at p < .01 under both estimators. ACS → CT remained significant under DWLS at p = .005. Estimator sensitivity was more consequential for the magnitude of the ACS-related paths than for their statistical significance.*

**S8. Institution Sensitivity Analyses**

Because only three institutions were included, multilevel SEM and institution-level cluster-robust inference were not considered reliable. Leave-one-institution-out and institution-specific models were therefore estimated. The focal coefficient remained positive and statistically significant in every specification.

**Table S8**

**Leave-One-Institution-Out and Institution-Specific PNT–LOU Estimates**

Panel A. Leave-one-institution-out analyses.

| **Excluded institution** | **Remaining N** | **β** | **SE** | **p** | **CFI** | **RMSEA** |
| --- | --- | --- | --- | --- | --- | --- |
| Institution A | 673 | .576 | .045 | < .001 | .985 | .025 |
| Institution B | 698 | .550 | .043 | < .001 | .988 | .021 |
| Institution C | 637 | .581 | .045 | < .001 | .989 | .021 |

Panel B. Institution-specific analyses.

| **Institution** | **N** | **β** | **SE** | **p** | **CFI** | **RMSEA** |
| --- | --- | --- | --- | --- | --- | --- |
| Institution A | 331 | .552 | .060 | < .001 | .993 | .016 |
| Institution B | 306 | .606 | .068 | < .001 | .980 | .029 |
| Institution C | 367 | .555 | .060 | < .001 | .985 | .025 |

Note. These checks indicate that the focal association was not attributable exclusively to one institution, but they do not eliminate all possible within-institution dependence.

**S9. Item-Level Descriptive Statistics, Standardised Loadings, and Standard Errors**

**Table S9**

*Item-Level Descriptive Statistics, Standardised Factor Loadings, Standard Errors, and Error Variances*

| Construct | Item | M | SD | Skewness | Kurtosis | Loading | SE | Error variance |
| --- | --- | --- | --- | --- | --- | --- | --- | --- |
| PNT | PTM1 | 3.828 | 0.842 | −0.248 | −0.505 | 0.760 | .016 | 0.422 |
| PNT | PTM2 | 3.897 | 0.840 | −0.350 | −0.426 | 0.743 | .017 | 0.448 |
| PNT | PTM3 | 3.768 | 0.913 | −0.367 | −0.402 | 0.766 | .016 | 0.414 |
| PNT | PTM4 | 3.865 | 0.83 | −0.350 | −0.332 | 0.755 | .017 | 0.430 |
| PNT | PTM5 | 3.941 | 0.803 | −0.356 | −0.309 | 0.720 | .018 | 0.482 |
| LOU | LOU1 | 3.958 | 0.819 | −0.371 | −0.497 | 0.698 | .020 | 0.513 |
| LOU | LOU2 | 3.963 | 0.790 | −0.336 | −0.386 | 0.716 | .019 | 0.488 |
| LOU | LOU3 | 3.871 | 0.841 | −0.216 | −0.674 | 0.697 | .020 | 0.515 |
| LOU | LOU4 | 3.759 | 0.891 | −0.306 | −0.477 | 0.728 | .018 | 0.470 |
| LOU | LOU5 | 3.963 | 0.810 | −0.350 | −0.456 | 0.708 | .019 | 0.499 |
| ACS | NS1 | 3.828 | 0.843 | −0.325 | −0.389 | 0.758 | .017 | 0.426 |
| ACS | NS2 | 3.754 | 0.883 | −0.222 | −0.540 | 0.729 | .018 | 0.468 |
| ACS | NS3 | 3.753 | 0.874 | −0.324 | −0.343 | 0.731 | .018 | 0.466 |
| ACS | NS4 | 3.742 | 0.877 | −0.308 | −0.370 | 0.716 | .018 | 0.487 |
| ACS | NS5 | 3.830 | 0.867 | −0.253 | −0.604 | 0.772 | .016 | 0.404 |
| SRL | SRL1 | 3.949 | 0.778 | −0.320 | −0.394 | 0.729 | .018 | 0.468 |
| SRL | SRL2 | 3.898 | 0.826 | −0.299 | −0.455 | 0.713 | .019 | 0.491 |
| SRL | SRL3 | 3.824 | 0.862 | −0.382 | −0.164 | 0.729 | .018 | 0.468 |
| SRL | SRL4 | 3.893 | 0.812 | −0.317 | −0.346 | 0.731 | .018 | 0.465 |
| SRL | SRL5 | 3.857 | 0.797 | −0.317 | −0.161 | 0.704 | .019 | 0.505 |
| CT | CT1 | 3.768 | 0.890 | −0.276 | −0.441 | 0.734 | .018 | 0.462 |
| CT | CT2 | 3.661 | 0.933 | −0.246 | −0.568 | 0.750 | .017 | 0.438 |
| CT | CT3 | 3.789 | 0.868 | −0.340 | −0.312 | 0.745 | .017 | 0.445 |
| CT | CT4 | 3.710 | 0.923 | −0.286 | −0.473 | 0.749 | .017 | 0.439 |
| CT | CT5 | 3.755 | 0.910 | −0.350 | −0.398 | 0.720 | .018 | 0.482 |

*Note. Loadings are standardised estimates, and SE denotes the corresponding standard error. All standardised factor loadings were statistically significant at p < .001. The measurement model was estimated from item-level data without item parcels, cross-loadings, or correlated residuals.*

**S10. Latent Correlations and HTMT Ratios**

**Table S10**

*Latent Correlations and HTMT Ratios for the Five Focal Constructs*

| Construct | PNT | LOU | ACS | SRL | CT |
| --- | --- | --- | --- | --- | --- |
| PNT | — | .534 | .467 | .384 | .345 |
| LOU | .534 | — | .477 | .474 | .410 |
| ACS | .469 | .479 | — | .463 | .383 |
| SRL | .382 | .474 | .463 | — | .525 |
| CT | .345 | .411 | .383 | .524 | — |

*Note. Values above the diagonal are standardised latent-factor correlations from the five-factor measurement model. Values below the diagonal are heterotrait–monotrait (HTMT) ratios calculated from the item-level data. All HTMT values were below .85, with a maximum of .534.*

**S11. Additional Measurement-Quality Indicators**

The five-factor model was estimated without correlated residuals, cross-loadings, or item parcels. The mean absolute residual correlation was .016, no residual correlation exceeded |.10|, and the largest residual was −.066. The largest modification index was 9.26 and concerned a potential cross-loading of LOU2 on PNT; this parameter was not freed in the reported model. Factor score determinacy exceeded .98 for every construct. These diagnostics support construct separability within the specified measurement model but do not rule out common-method inflation.

**Table S11**

*Additional Measurement-Quality Indicators*

| **Construct** | **Factor determinacy** | **AVE** | **CR** | **Cronbach’s α** |
| --- | --- | --- | --- | --- |
| PNT | .996 | .561 | .865 | .864 |
| LOU | .982 | .503 | .835 | .835 |
| ACS | .995 | .550 | .859 | .859 |
| SRL | .995 | .520 | .844 | .844 |
| CT | .997 | .547 | .858 | .858 |

*Note. Composite reliability was calculated from standardised factor loadings and corresponding error variances. The close similarity between α and CR reflects the relatively homogeneous loading patterns across the five-item measures.*

**S12. PNT Dimensionality Sensitivity Analysis**

Because the five PNT items represent several potentially distinguishable manifestations of teacher-referenced normative-positioning difficulty, alternative one-factor and two-factor specifications were compared. In the two-factor specification used for this sensitivity analysis, PTM1–PTM3 represented students’ perceived disagreement or uncertainty about alignment with teacher-referenced expectations, whereas PTM4–PTM5 represented lack of clarity or inconsistency in those expectations. The comparison was treated as a sensitivity analysis rather than as definitive scale-development evidence.

**Table S12**

*Alternative Factor Specifications for Perceived Teacher-Referenced Normative Tension*

| **Model** | **χ²** | **df** | **p** | **CFI** | **TLI** | **RMSEA** | **SRMR** | **Latent correlation** |
| --- | --- | --- | --- | --- | --- | --- | --- | --- |
| One-factor PNT | 12.00 | 5 | .035 | .997 | .993 | .037 | .015 | — |
| Constrained two-factor | 6.94 | 5 | .225 | .999 | .998 | .020 | .013 | .950 |
| Unconstrained two-factor | 3.63 | 4 | .459 | 1.000 | 1.000 | .000 | .008 | .949 |

Nested comparison: one-factor versus unconstrained two-factor, Δχ²(1) = 8.37, p = .004.

*Note. In the two-factor specifications, Factor 1 included PTM1–PTM3 and Factor 2 included PTM4–PTM5. Factor 1 represented perceived disagreement or uncertainty about alignment with teacher-referenced expectations; Factor 2 represented unclear or inconsistent expectations. Although the unconstrained two-factor model improved fit relative to the one-factor model, the estimated factor correlation was .949, indicating extremely limited empirical separation between the two proposed dimensions. The decision to retain a single PNT factor was therefore based on parsimony and the very high interfactor correlation rather than on evidence that the one-factor representation was uniquely superior. The two proposed dimensions were not interpreted separately in the present study. Values of CFI and TLI slightly exceeding 1.000 were reported as 1.000.*

**S13. Internal Split-Sample Measurement Analysis**

The final student sample was randomly divided into calibration and validation subsamples of equal size using a fixed random seed of 20260719. Parallel analysis and principal-axis exploratory factor analysis with oblimin rotation were conducted in the calibration subsample. The five-factor representation was subsequently evaluated through confirmatory factor analysis in the held-out validation subsample.

**Table S13a**

*Parallel Analysis in the Calibration Subsample*

| **Component** | **Observed eigenvalue** | **95th-percentile random eigenvalue** | **Retain** |
| --- | --- | --- | --- |
| 1 | 7.886 | 1.488 | Yes |
| 2 | 2.511 | 1.409 | Yes |
| 3 | 2.085 | 1.354 | Yes |
| 4 | 1.875 | 1.302 | Yes |
| 5 | 1.631 | 1.262 | Yes |
| 6 | 0.645 | 1.227 | No |

*Note. Calibration subsample n = 502. Random eigenvalues represent the 95th percentiles obtained from 300 within-item permutations. Five factors were retained because the first five observed eigenvalues exceeded the corresponding random eigenvalues.*

The complete pattern matrix is reported to permit inspection of cross-loadings. Numerical factor correlations were not used as criteria for retaining the five-factor solution; retention was based on parallel analysis, loading pattern, and held-out CFA.

**Table S13b**

*Oblimin-Rotated Exploratory Factor Loadings in the Calibration Subsample*

| **Item** | **Factor_1** | **Factor_2** | **Factor_3** | **Factor_4** | **Factor_5** |
| --- | --- | --- | --- | --- | --- |
| PTM1 | .730 | .059 | .069 | −.044 | .001 |
| PTM2 | .748 | −.023 | .025 | .006 | −.014 |
| PTM3 | .731 | −.023 | −.006 | .050 | −.004 |
| PTM4 | .753 | .010 | −.006 | .016 | .006 |
| PTM5 | .656 | .041 | −.018 | −.019 | .117 |
| LOU1 | −.035 | .016 | .063 | −.001 | .705 |
| LOU2 | .121 | −.045 | −.027 | .068 | .673 |
| LOU3 | .092 | .048 | −.045 | .109 | .594 |
| LOU4 | −.014 | .017 | .000 | .005 | .714 |
| LOU5 | −.013 | .022 | .052 | −.051 | .734 |
| NS1 | .028 | .014 | .807 | .020 | −.081 |
| NS2 | −.008 | −.023 | .707 | −.018 | .109 |
| NS3 | −.009 | −.014 | .782 | −.005 | .005 |
| NS4 | .094 | −.004 | .683 | .011 | .009 |
| NS5 | −.039 | .033 | .748 | .029 | .031 |
| SRL1 | −.027 | .101 | .047 | .693 | −.012 |
| SRL2 | −.064 | .000 | .026 | .731 | −.007 |
| SRL3 | .016 | .010 | .000 | .715 | .026 |
| SRL4 | .076 | −.052 | −.004 | .725 | −.002 |
| SRL5 | .000 | −.010 | −.023 | .701 | .033 |
| CT1 | .077 | .741 | −.053 | .015 | −.016 |
| CT2 | −.065 | .751 | .030 | −.036 | .094 |
| CT3 | .045 | .713 | −.003 | .013 | .004 |
| CT4 | .039 | .755 | .026 | .016 | −.096 |
| CT5 | −.059 | .687 | .021 | .049 | .057 |

*Note. Calibration subsample n = 502. Principal-axis factoring with oblimin rotation was used. The table reports the complete loading matrix for all 25 indicators across all five extracted factors; absolute loadings of .30 or greater are substantively salient. Factor labels were assigned after inspection of item content, and the order of extracted factors does not imply theoretical priority. Because oblimin rotation permits correlated factors, the solution should not be interpreted as orthogonal.*

**Table S13c**

*Confirmatory Factor Analysis in the Held-Out Validation Subsample*

| **Fit index** | | **Value** | |
| --- | --- | --- | --- |
| N | | 502 | |
| χ² | | 269.02 | |
| df | | 265 | |
| p | | .420 | |
| CFI | | .999 | |
| TLI | | .999 | |
| RMSEA | | .006 | |
| SRMR | | .030 | |
| **Item** | **Construct** | | **Standardised loading** |
| PTM1 | PNT | | .757 |
| PTM2 | PNT | | .746 |
| PTM3 | PNT | | .802 |
| PTM4 | PNT | | .747 |
| PTM5 | PNT | | .717 |
| LOU1 | LOU | | .682 |
| LOU2 | LOU | | .686 |
| LOU3 | LOU | | .691 |
| LOU4 | LOU | | .752 |
| LOU5 | LOU | | .696 |
| NS1 | ACS | | .724 |
| NS2 | ACS | | .715 |
| NS3 | ACS | | .688 |
| NS4 | ACS | | .696 |
| NS5 | ACS | | .774 |
| SRL1 | SRL | | .704 |
| SRL2 | SRL | | .719 |
| SRL3 | SRL | | .722 |
| SRL4 | SRL | | .741 |
| SRL5 | SRL | | .708 |
| CT1 | CT | | .719 |
| CT2 | CT | | .748 |
| CT3 | CT | | .755 |
| CT4 | CT | | .752 |
| CT5 | CT | | .718 |

*Note. The confirmatory model specified five correlated latent factors corresponding to PNT, LOU, ACS, SRL, and CT. No item parcels or correlated residuals were used. Because the calibration and validation subsamples originated from the same full sample, this analysis represents internal replication rather than independent external validation.*

**S14. Exploratory Structural Extensions**

Three nested structural specifications were compared to assess whether the theory-constrained six-path model exhausted the observed covariance structure. The focal model contained the six theoretically specified paths. The second model added a direct path from PNT to ACS, and the third model additionally added direct paths from PNT to SRL and CT. These additions were exploratory, were not treated as confirmatory hypothesis tests, and were used to evaluate the structural limits of the focal specification.

**Table S14a**

*Fit of the Primary and Exploratory Structural Models*

| **Model** | **χ²** | **df** | **CFI** | **TLI** | **RMSEA** | **SRMR** | **Comparison** |
| --- | --- | --- | --- | --- | --- | --- | --- |
| Primary model | 400.03 | 269 | .988 | .986 | .022 | .055 | — |
| + PNT→ACS | 347.16 | 268 | .993 | .992 | .017 | .045 | Δχ² = 52.87, Δdf = 1, p < .001 |
| + PNT→ACS, SRL, CT | 338.11 | 266 | .993 | .992 | .016 | .044 | Δχ² = 9.05, Δdf = 2, p = .011 |

**Table S14b**

*Standardised Coefficients Across the Primary and Exploratory Structural Models*

| **Outcome** | **Predictor** | **Primary model β** | **Add PNT→ACS β** | **Fully expanded model β** |
| --- | --- | --- | --- | --- |
| LOU | PNT | .568*** | .544*** | .535*** |
| ACS | LOU | .504*** | .311*** | .318*** |
| ACS | PNT | — | .304*** | .297*** |
| SRL | LOU | .361*** | .349*** | .303*** |
| SRL | ACS | .292*** | .312*** | .286*** |
| SRL | PNT | — | — | .092* |
| CT | LOU | .331*** | .318*** | .268*** |
| CT | ACS | .230*** | .250*** | .221*** |
| CT | PNT | — | — | .103* |

Adding PNT→ACS substantially improved model fit. The PNT→LOU coefficient remained positive and sizeable, whereas the LOU→ACS coefficient decreased from .504 to .311 and the PNT→ACS coefficient was .304. These results indicate that the six-path model is a theory-constrained focal specification rather than a structurally exhaustive account. In particular, they do not support interpreting the six-path specification as fully accounting for the association between PNT and ACS.

*Note. Coefficients are standardised. The primary model contains only the six theoretically specified paths. Additional PNT direct paths were exploratory. *p < .05. ***p < .001. The significant exploratory paths do not establish temporal or causal effects.*

**Table S14c**

*Standardised Estimates for the Primary Six-Path Structural Model*

| **Path** | **β** | **SE** | **p** |
| --- | --- | --- | --- |
| PNT → LOU | .568 | .036 | < .001 |
| LOU → ACS | .504 | .045 | < .001 |
| LOU → SRL | .361 | .043 | < .001 |
| LOU → CT | .331 | .050 | < .001 |
| ACS → SRL | .292 | .036 | < .001 |
| ACS → CT | .230 | .043 | < .001 |

*Note. β = standardised structural coefficient; SE = standard error. Estimates are from the theoretically specified six-path model.*
